# Optical CAD Utilization for the Design and Testing of a LED Streetlamp

**DOI:** 10.3390/ma10090985

**Published:** 2017-08-24

**Authors:** David Jafrancesco, Luca Mercatelli, Daniela Fontani, Paola Sansoni

**Affiliations:** CNR-INO National Institute of Optics, Largo E. Fermi, 6-50125 Firenze, Italy; david.jafrancesco@ino.it (D.J.); luca.mercatelli@ino.it (L.M.); daniela.fontani@ino.it (D.F.)

**Keywords:** lighting, optical design, streetlamp, LED source

## Abstract

The design and testing of LED lamps are vital steps toward broader use of LED lighting for outdoor illumination and traffic signalling. The characteristics of LED sources, in combination with the need to limit light pollution and power consumption, require a precise optical design. In particular, in every step of the process, it is important to closely compare theoretical or simulated results with measured data (obtained from a prototype). This work examines the various possibilities for using an optical CAD (Lambda Research *TracePro*) to design and check a LED lamp for outdoor use. This analysis includes the simulations and testing on a prototype as an example; data acquired by measurement are inserted into the same simulation software, making it easy to compare theoretical and actual results.

## 1. Introduction

In order to better substitute traditional light sources (halogen, fluorescent or High-Intensity-Discharge lamps) with LED (light-emitting diode) sources, lamp design must include a soundness phase, as traditional sources emit a radiation lobe of 4π steradian while white LED sources emit a radiation lobe of 2π steradian or less. Considering that the substitution process strongly depends both on costs and performance reliability, it has been clear for many years that the use of optical simulation software is indispensable, as it allows enhancement and refinement of the design activity [1]. On the other hand, it is necessary for the designer to be very confident in the comparison between simulated and measured results, in order to validate the simulated lamp model and modify or improve it without a large number of prototypes. This work highlights a method of designing and testing a streetlamp using optical CAD (computer-aided design) software, in particular looking for high illuminance uniformity values on the ground. The methodology involves reverse raytracing starting from the resultant illumination in order to obtain information on the source design. The distinctive trait of this streetlamp is that it utilizes only reflective surfaces, because the utilization of refraction optics (plastic lenses) involves risks concerning their reliability [2,3]. Some studies have been performed in order to manufacture glass-molded lenses for streetlamps: H. Kočárkova, in [4], shows that a streetlamp equipped with a glass lens produced good results, but some problems with shape control remain [5]. Thus, the proposed method is applied to the design and testing of a streetlamp that could be utilized in different situations with few adjustments (such as the LED current or the component inclination), whose optical components are SMD (surface-mounted device) white LEDs and reflective parabolic surfaces. The constraints, requirements and optical design of the LED streetlamp have already been described in a previous work [6].

## 2. Software Tool

The choice of optical CAD software is necessary to proceed to the design phase (see for example [7,8,9]; the latter utilizes the same commercial software, Lambda Research TracePro (Lambda Research Corporation, Littleton, MA, USA), used to perform the simulations reported in this paper). Using traditional sequential optical design software (like Code V by Synopsys or OpticStudio—Zemax by Zemax) is not a good choice, because needs the sequence in which the rays hit the lamp surfaces to be defined. Thus, non-sequential software has to be used, as highlighted in a previous study [8] that utilizes Monte-Carlo raytracing. In this case, the rays can hit whichever surface, because the software calculates the behavior of the single ray taking into account its actual path (a valuable paper, though quite old, that summarizes the advantages of non-sequential raytracing is [1]). Moreover, optical CAD software permits us to define the optical characteristics of the internal surfaces of the streetlamp. Many software tools provide a BSDF (bidirectional scattering distribution function) implementation in order to take into account diffuse reflection and transmission, although in many cases the ability to simulate a partial Lambertian diffusion could be sufficient. Other important features of optical CADs are the functions to export data and to import files from mechanical CAD (e.g., STEP or IGES files) or measurement data tables. In particular, the data can be exported in easy and immediate formats, such as illuminance maps or luminous intensity plots (although the elaboration time in order to obtain a map could be significant). Moreover, a key characteristic of the software is the ability to interact with the designer/tester during all the phases of development. Hence, the main software capacities needed to manage the design of a streetlamp can be summarized as follows:

- Non-sequential raytracing
- Simulation of diffusing, reflecting or transmitting surfaces
- Ability to import data from mechanical CADs
- Ability to output data as illuminance maps on the ground and luminous intensity maps
- Generation of a luminous source from external data (from experimental measurements)

### 2.1. Reference System for the Example Streetlamp

The area to be illuminated on the ground was defined as a 30 m × 10 m rectangle (the last value is the road width); the street lamp is placed at a height of 8 m from the ground, equally distant from the two short sides of the rectangle, near the long side (the projection of the lamp on the ground is inside the rectangle, located 1 m far away from the nearest border). A better description of the initial data is included in [6].

The adopted Reference System is described as follows:

- Y-axis is vertical (positive upwards);
- X-axis is parallel to the roadway;
- Z-axis is perpendicular to the road side (positive direction from the side of the road towards the center of the road);
- The origin corresponds to the projection on the ground of the center of the outer edge of the lamp (the side closest to the road border).

**Figure 1:**
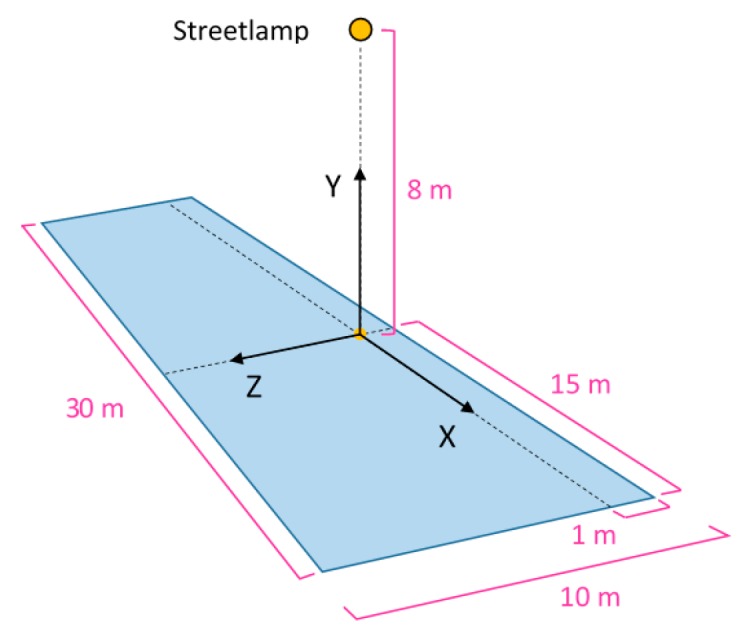
Outline of the streetlamp position and the Reference System.

In Figure 1 an outline of the streetlamp position and the Reference System is shown.

### 2.2. Optical Design

**Figure 2:**
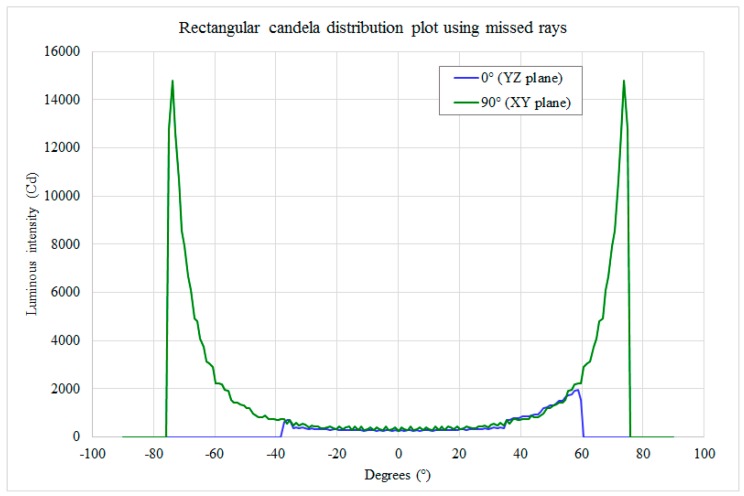
Theoretical candela emission in order to obtain a uniform illumination of the street, in blue along the street short side (YZ plane, angles > 0 from negative Y direction towards the center of the road), in green along its long side (YX plane).

The optical CAD can be utilized first in the definition of the lamp’s angular lobe of emission. The requirements involved in streetlamp performance tests require the evaluation of luminance, then they take into account the reflectance characteristics of the road surface (it is not a Lambertian reflector). Due to the fact that the lamp is not designed for a single road or a specific asphalt type, luminous intensity or illuminance data are utilized to characterize the lamp [4,5,10,11]. The goal is to realize a uniform illumination of the street, reaching a minimum level of lux at every point. Once the dimensions of the illuminated street and the position of the lamp are defined, in the optical CAD it is possible to set a source with the same size and position on the street, assign to it a spatial uniform emission (5 lm/m^2^) and impose that rays from the source are directed toward the center of the lamp. In TracePro, the simplest method to do this is by means of a “grid source”; generally, for non-uniform illuminated areas or in other optical CADs it is possible to generate a “file source” with start position, direction and flux of all the rays. The result is a set of rays that defines a radiation lobe (candela plot); if the streetlamp emits the same radiation lobe (obviously reversed), the result is a uniform illumination of the street. The rectangular candela plot of rays from the street to the center of the lamp is shown in Figure 2. However, the optical designer is more interested in the radiation lobe (angular map) with respect to street illumination data (ground map), because the first one is directly related to the lamp design and more easily measurable.

In our case study, the request was to use only reflective surfaces: a standard choice to avoid problems with yellowing of plastic lenses [2,3]. Then, a part of the light emitted by the LEDs has to be reflected while the other part directly illuminates the road; the aim is to have both high efficiency and uniform distribution of irradiance on the ground. The CPC (Compound Parabolic Concentrator) was chosen as a reflector (see ([12] pp. 50–64) for a description of the CPC geometry and characteristics); please note that this kind of optical device is generally implemented in optical CADs. The standard use of the CPC is to concentrate the light on a detector surface, but due to its optical characteristics it permits efficient management of the rays (in particular of the radiation lobe) when a source is mounted in place of the detector because:

- All the rays that start from or cross the smaller window of the CPC exit from the large window, then the efficiency of the device is theoretically of 100% (excluding the surface absorption); and
- The ray inclination is joined to the starting or crossing point of the ray.

Moreover, it is easier to make parabolic surfaces with respect to free-form optics, which is sometimes used for these applications [13]. The utilization of a CPC to increase the illumination uniformity is briefly suggested in reference [14] (p. 328).

Each CPC manages the light generated by three SMD white LEDs (mounted aligned on the same printed circuit) and illuminates a defined zone on the ground; each SMD white LED was simulated as a disk with radius 2.6 mm (size of Cree XP-G LEDs, see [15]) that emits 90 lm from its upper surface. Its emission lobe was inserted into the optical CAD source database.

**Figure 3:**
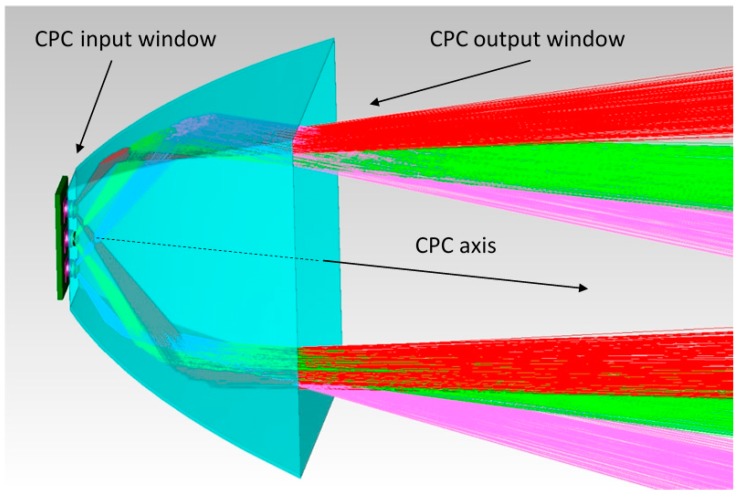
Behavior of the CPC.

Figure 3 illustrates the behavior of the CPC (sketched as semi-transparent) with three LEDs placed on its input window: please note that the output ray inclination is the same for rays emitted by an LED with equal inclination that are reflected on the opposite CPC surfaces.

**Figure 4:**
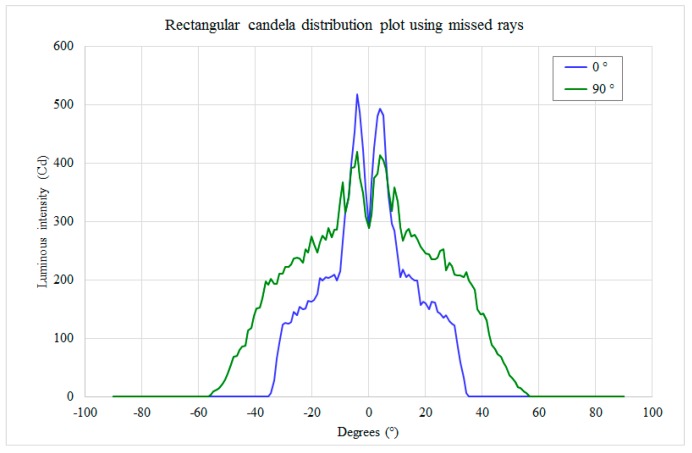
CPC candela emission, in blue, on the plane that contains the CPC axis; it is parallel to the long side of the CPC output window, in green, on the plane that contains the CPC axis and to the short side of the CPC output window.

The reflector parameters were set in order to obtain a radiation lobe narrow enough to illuminate the extreme zones of the street and large enough to realize a uniform illumination. Taking into account the size of the lamp, the planarity of the external glass and the need to utilize a unique CPC configuration, the best CPC shape generates the radiation lobe in Figure 4.

**Figure 5:**
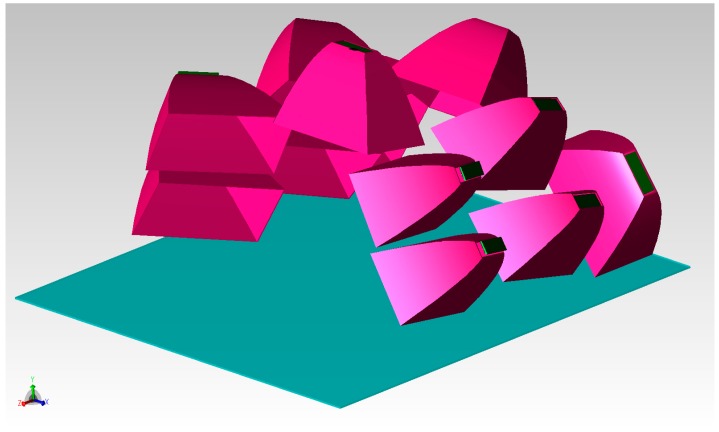
Render of the “compact streetlamp”, 3/4 view.

The trade-off between the lamp’s simplicity and good illumination on the ground was defined, for the actual design, using 10 side reflectors (5 for each side, utilized to light the more distant zones) and 2 central reflectors (to illuminate the street under the lamp). All the reflectors have equal truncated pyramidal shape and parabolic surfaces; the size of their output window is 110 mm × 58 mm, and their length is 50 mm. Each reflector manages the light emitted by a strip of three Cree XLamp XP-G SMD LEDs; the strip is placed at the input window of the reflector, and the LEDs are placed 12 mm from each other. Considering the flux values of these LEDs (see the datasheet [15]), the emission of each LED was set to 90 lm (the decrement of the lumen tabulated value due to the expected maximum junction temperature of 85 °C had already been taken into account). Taking into account Figure 2 and Figure 4, the CPCs were “assembled” to compose the whole lamp shown in Figure 5. This is the chosen configuration, called “compact streetlamp”. The lamp is composed of an external case, measuring 600 mm × 600 mm × 200 mm, with a flat protection glass toward the ground.

**Figure 6:**
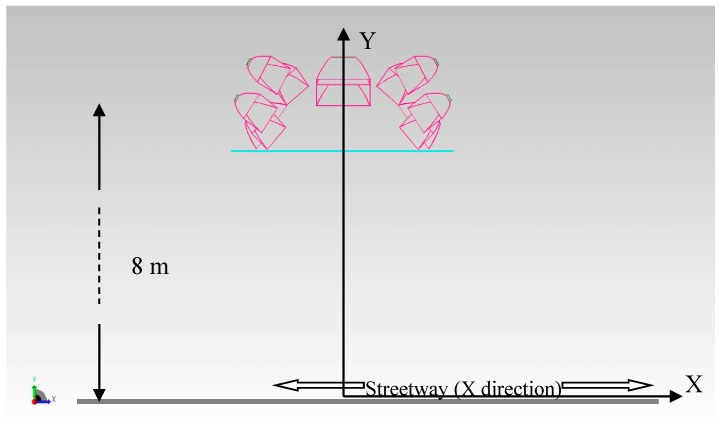
Frontal view (silhouette) from the opposite side of the streetway.

The scheme in Figure 6 illustrates the position of the compact streetlamp with respect to the street.

The lamp configuration is obviously symmetrical with respect to the YZ-plane, which is perpendicular to the road direction.

**Figure 7:**
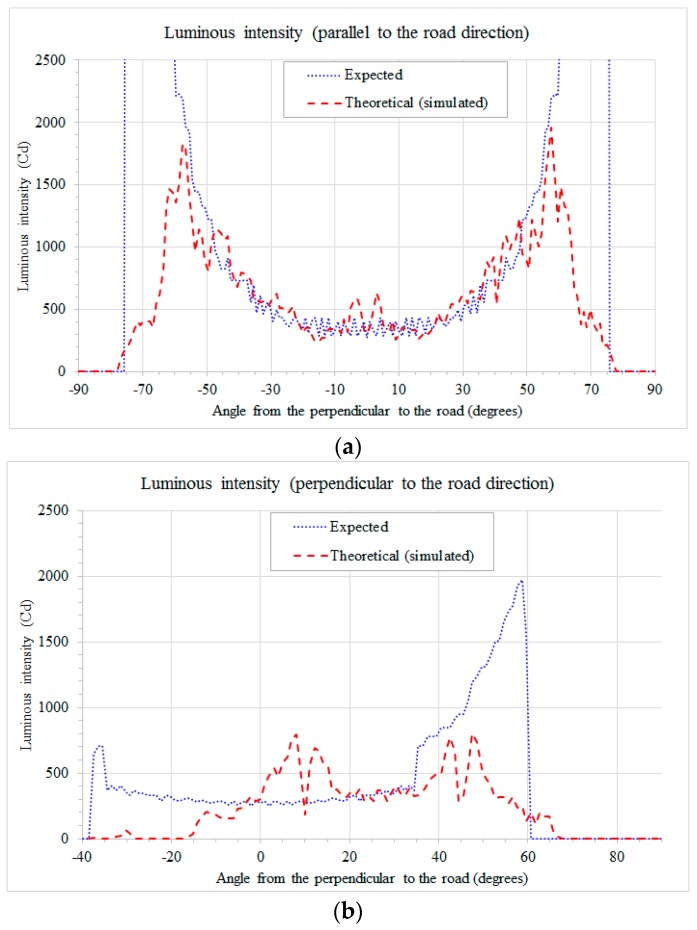
(**a**) Comparison between expected and theoretical (simulated) data of the luminous intensity parallel to the road direction (YX plane); (**b**) comparison between expected and theoretical (simulated) data of the luminous intensity perpendicular to the road direction (YZ plane, angles > 0 from negative Y direction towards the center of the road).

The results of the simulation (rectangular candela plot) for the modeled compact streetlamp are shown in Figure 7a,b, where they are indicated as theoretical (simulated), in red color. The profile parallel to the road direction is on the XY-plane, while the profile perpendicular to the road direction is on the YZ-plane. As expected, the profile along the road direction is symmetrical while the profile perpendicular to the road direction is asymmetrical.

To simulate the actual road illumination, three compact streetlamps were considered for the lighting simulation, placed on the same side of the road with a 30 m gap. The detector is a surface that simulates a piece of road with size 30 m × 10 m; the central streetlamp is placed in the point of (x, y, z) coordinates (0, 8 m, 0), the top-left and right-down corners of the piece of road are (−15 m, 0, −1 m) and (15 m, 0, 9 m). The coordinates of the two other streetlamps are (−30 m, 8 m, 0) and (30 m, 8 m, 0), and they contribute to illuminate the lateral sides of the simulated piece of road that works as detector.

**Figure 8:**
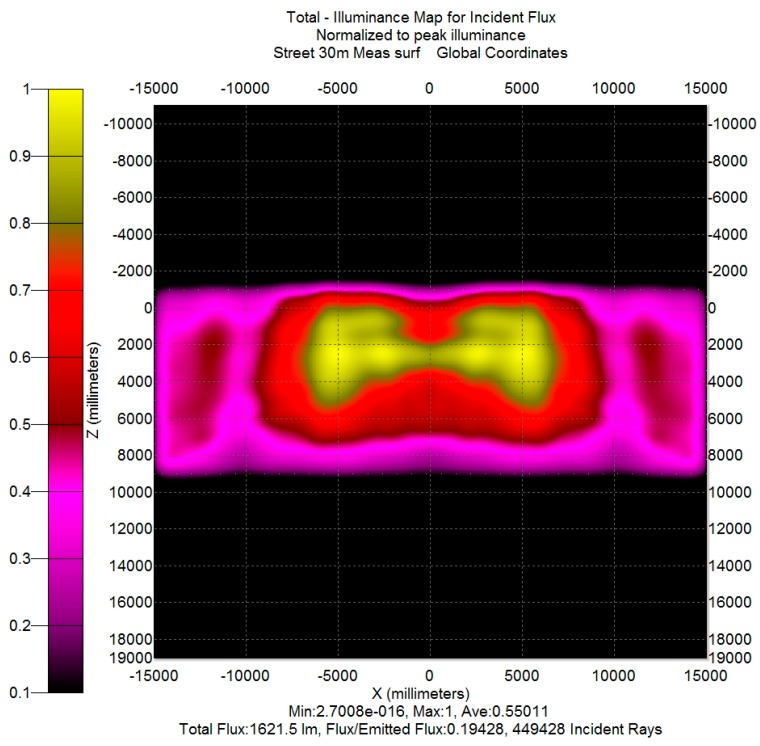
Theoretical (simulated) illuminance map on the ground for the compact streetlamp.

The illuminance map of the piece of road described above is reported in Figure 8 (in order to permit a better comparison with the following results, the illuminance is scaled on its maximum). Obviously, the map refers to the three streetlamps, but in order to evaluate the efficiency of a single streetlamp, it could be concluded that the light from the side streetlamps that falls into the detector is balanced by the light generated by the central streetlamp that falls into the zones under the side streetlamps. Thus, the efficiency of a single streetlamp can be evaluated as the flux on the simulated road surface divided by the total flux generated by the streetlamp: its value is 0.56. The uniformity E_avr_/E_min_ is about 2, a value within the limits reported in [16] (p. 22-10, Figure 22-8b).

The prototype was manufactured according to the layout of the simulated model. The optical part (reflector and LED stripes) was sealed in order to prevent oxidation phenomena.

**Figure 9:**
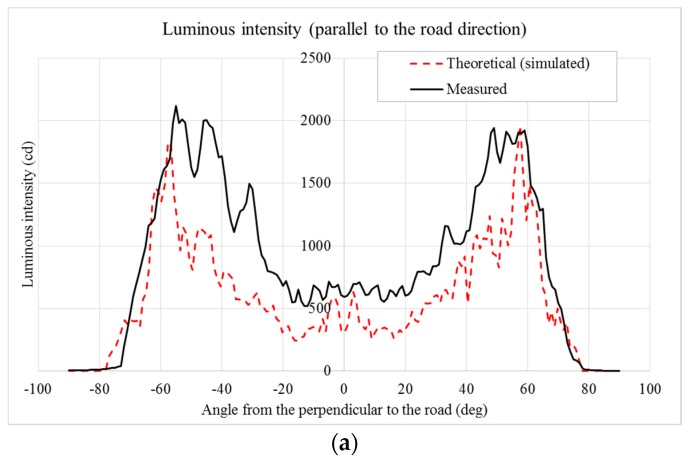

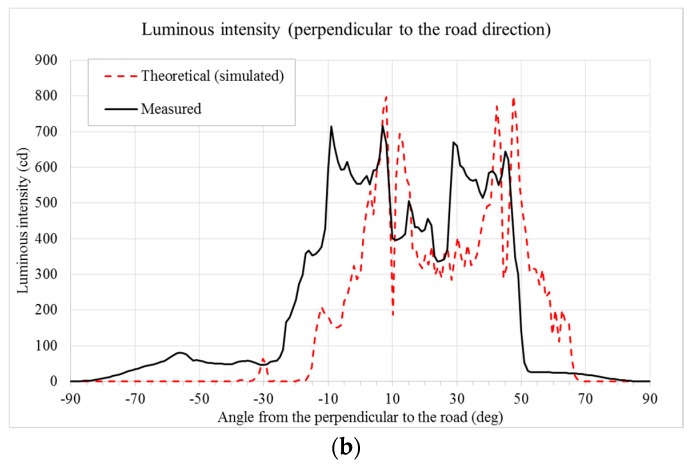
(**a**) Comparison between theoretical (simulated) and measured data of the luminous intensity parallel to the road direction; (**b**) comparison between theoretical (simulated) and measured data of the luminous intensity perpendicular to the road direction.

The emission lobe of the prototype was measured by means of a System A gonio-photometer (the gonio-photometer system types are described in [17], pp. 4–9). For the test on the prototype, for technical reasons, the LED current was limited to 75% of the nominal value, and in this case the light output of each LED is estimated to be about 67.5 lm. The results are shown in Figure 9a (luminous intensity in the direction parallel to the road) and Figure 9b (luminous intensity in the direction perpendicular to the road). These figures also report the simulated (theoretical) data, so it is easier to compare designed and experimental data.

Please note that the measured data are very similar to the theoretical (simulated) data for the direction parallel to the road (Figure 9a), while there is a shift of the inclination (toward the vertical axis) of the measured data in the direction perpendicular to the road (Figure 9b).

**Figure 10:**
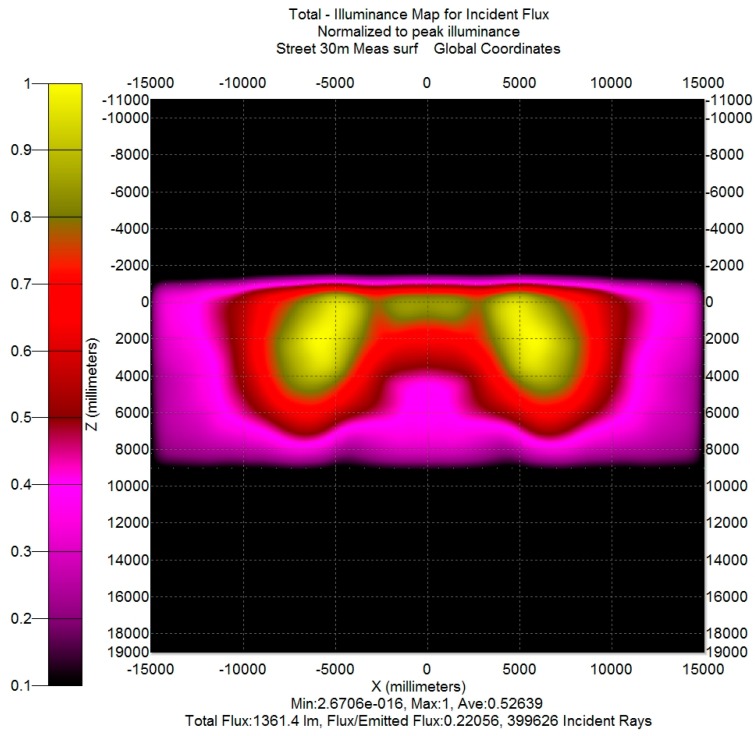
Experimental (simulated) illuminance map on the road for the prototype of the compact streetlamp.

In order to obtain a data set usable for TracePro, the set of measured data had to be transformed in a Eulumdat file (the Eulumdat format is a de-facto industry standard and not defined by any official technical report; a description of its format is available on the web site www.helios32.com), that nevertheless requires the output data from a System C gonio-photometer. Thus, the preliminary passage is the transformation of the System A gonio-photometer output into a System C gonio-photometer output by applying the transformation rules contained in reference [17] (p. 9), and then a Eulumdat file was created by means of a VBA (Visual Basic for Application) procedure. This file was imported into TracePro in order to create a Surface Source lobe (i.e., a radiation lobe, defined from the luminous intensity data specified in the Eulumdat file). In the TracePro model, that radiation lobe can be utilized only if it is applied to a specific surface; the chosen surface was the output surface of the exterior glass of each simulated streetlamp. That allows us to easily compare, by simulation, the “theoretical” model (the result of the lamp design phase) and an “experimental” model (obtained from the measurements on the prototype as illustrated above). For the example case, the comparison between Figure 8 and Figure 10 shows that there is a difference between theoretical (simulated) results and experimental (simulated) results. In particular:

- The actual light distribution (Figure 10) on the lateral sides of the rectangle is not as uniform as the theoretical (simulated) one (Figure 8).
- Opposite to the lamp, on the other side of the street (near the point X = 0, Z = 6000 mm), there is a lack of illuminance (highlighted by the purple color instead of red color) on the map reported in Figure 10 with respect to the map in Figure 8.

**Figure 11:**
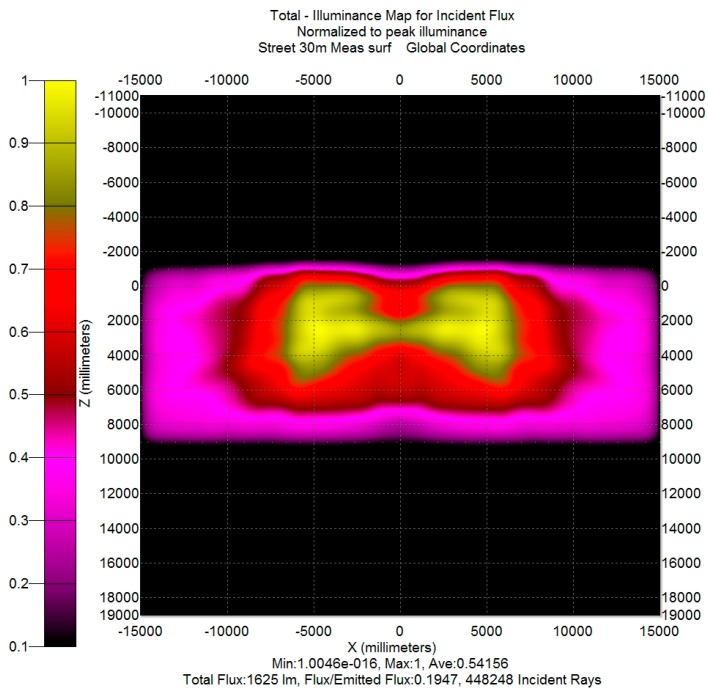
Theoretical (simulated) illuminance map on the road for the compact streetlamp with LEDs position of the CPCs modified.

In the case study, the first difference was seen to be due to the incorrect placement of the LEDs for some CPCs in the prototype. The reason for the second difference was unclear, due to the fact that in the prototype the external glass was sealed with the CPC plate (which was whole), which means the optical characteristics of the CPCs were not measurable. It is conceivable that the second difference is due to an incorrect mode of operation of the two central CPCs. However, in order to partially control the correspondence between theoretical results and experimental ones, the theoretical model of the streetlamp was changed to take into account the different position of the printed circuit for the modified CPCs. The corresponding illuminance map obtained by simulation is shown in Figure 11.

**Figure 12:**
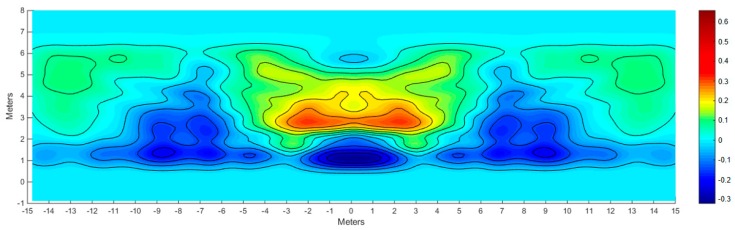
Difference between theoretical (simulated) illuminance map (Figure 11) and experimental (simulated) illuminance map (Figure 10).

The new theoretical (simulated) illuminance map (Figure 11) is very similar to the experimental (simulated) illuminance map obtained from the actual measurement of the lamp (Figure 10), except for the illuminance difference on the street side opposite to the lamp. In order to highlight this difference, in Figure 12 the difference map (obtained subtracting Figure 10 from Figure 11) is shown. Due to the fact that the values on the illuminance maps are relative to their maximum, the difference map supplies information only about the uniformity.

Clearly the experimental results follow the theoretical ones in the lateral zones, while there is a relevant difference in the central zone (between −0.4 and +0.2, where +1 is the maximum value of each illuminance map); the proximity of the positive and negative zones suggests that the central CPCs inside the prototype could be incorrectly oriented.

## 3. Conclusions

Traditional lamps have been replaced with LED-based lamps for outdoor illumination and traffic signaling also due to the recent update of the specific norms and regulations. However, these replacements always require optical design and testing as new light sources, in order to respect the norms and fulfill the requirements of every particular case. 

The optical design phase is essential to optimize LED lamp characteristics, minimize power consumption and reduce light pollution. The phase of experimentation on the prototypes is also fundamental to improving the manufacturing process of the LED device.

The combination of these two phases is the most delicate and constructive point. In every step of the process, the theoretical simulated results must be compared with the data acquired by measurement of a lamp prototype.

Using an optical CAD (Lambda Research TracePro), this comparison is obtained as an immediately readable plot (irradiance map) and quantitative results (irradiance data), based on the measurements inserted into the software.

The results of the prototype check confirm the flexibility of actual optical non-sequential CADs and their importance for both the design and test phase of the realization of a lamp. In particular, reverse raytracing (from the surface to be lighted to the lamp) can be performed in order to obtain a theoretical radiation lobe for the lamp’s emission.

In the test phase, the measured data can be inserted into the optical CAD to generate a simulated emission and an illuminance map similar to the map obtained from theoretical simulations, which makes it easier to compare theoretical and actual results and improve the prototype in order to achieve the best results.
